# Navigating the nexus between British Columbia’s public consumption and decriminalization policies of illegal drugs

**DOI:** 10.1186/s12961-024-01150-6

**Published:** 2024-05-23

**Authors:** Farihah Ali, Justine Law, Cayley Russell, Jean-Francois Crépault, João Castel-Branco Goulão, Kurt Lock, Jürgen Rehm

**Affiliations:** 1 Institute for Mental Health Policy Research (IMHPR), Centre for Addiction and Mental Health (CAMH), 33 Ursula Franklin St., Toronto, ON M5S 2S1 Canada; 2 Ontario CRISM Node Team (OCRINT), Canadian Research Initiative in Substance Misuse (CRISM), Centre for Addiction and Mental Health (CAMH), Toronto, Canada; 3 Dalla Lana School of Public Health, University of Toronto, Toronto, Canada; 4 Communications and Partnerships, CAMH, Toronto, Canada; 5 The Intervention Service for Addictive Behaviors and Addictions (SICAD), Lisbon, Portugal; 6 BC Centre for Disease Control, Vancouver, BC Canada; 7 Department of Psychiatry, University of Toronto, Toronto, Canada; 8 PAHO/WHO Collaborating Centre, CAMH, Toronto, Canada; 9 WHO European Region Collaborating Centre at Public Health Institute of Catalonia, Barcelona, Spain; 10 Zentrum für Interdisziplinäre Suchtforschung der Universität Hamburg (ZIS), Universitätsklinikum Hamburg-Eppendorf, Hamburg, Germany

**Keywords:** Decriminalization, Canada, British Columbia, Substance use, Stigma, Public drug consumption, Bylaws

## Abstract

In January 2023, the province of British Columbia (BC) decriminalized the possession of certain illegal drugs for personal use. The province’s primary intent was to reduce the stigma associated with drug use, as well as barriers for people who use drugs (PWUD) to access treatment and supports. However, less than ten months into the decriminalization policy, due to growing concerns about public safety voiced by municipal governments and communities, the provincial government made amendments to the policy to ban the public consumption of illicit drugs in additional locations, and subsequently introduced additional legislation, *Bill 34*, aimed at regulating public consumption of drugs in public spaces. Some communities have also implemented local bylaws similarly regulating public drug use. *Bill 34* and local bylaws may serve as tools to promote community health and safety and minimize direct and indirect harms associated with public drug use. However, such legislation may re-criminalize PWUD and reinforce negative perceptions surrounding drug use, especially if these policies are not paired with strategies to expand the availability and accessibility of critical harm reduction and housing services. Without ample access to these services, limitations on public drug use can potentially displace individuals to areas where they are more likely to use alone, further exposing them to substance use-related harms, and undermining the goals of decriminalization. The potential effects of these restrictions may also disproportionately impact marginalized populations. As of April 2024, *Bill 34* remains on hold. Moving forward, it will be important to monitor this bill, as well as other public consumption bylaws and legislation, and their impact on BC’s overall decriminalization initiative. Decision-makers are urged to increase engagement with PWUD and relevant stakeholders in the design and implementation of policies pertaining to public consumption to ensure that they effectively address the evolving needs and realities of PWUD, and align with decriminalization goals.

## Introduction

On January 31, 2023, a 3-year provincial drug decriminalization policy was piloted in British Columbia (BC) as an exemption granted by Health Canada under section 56 of the *Controlled Drugs and Substances Act* (CDSA) [1]. This policy aims to eliminate criminal penalties for possession of up to a cumulative 2.5 g of select illegal drugs for personal use by adults (aged 18+), including cocaine/crack-cocaine, methamphetamine, ecstasy, and opioids such as heroin, morphine, and fentanyl [1]. The primary goal of the decriminalization policy, as stated by the BC government, is to reduce public stigma and reframe the discourse surrounding drug use as a public health issue rather than a criminal justice issue [2]. This policy aims to limit police interactions with people who use drugs (PWUD) and redirect them away from the criminal justice system by encouraging and supporting access to health and social services. Although criminal penalties for possession under this 2.5 g threshold are removed, possession of drugs in amounts over the threshold is still illegal. In addition, there are several stipulations under the policy in which possession of illegal drugs (of any amount) remains a criminal offense, and for which people are still subject to arrests and confiscation of their drugs. These include possession for purposes other than personal use (e.g., trafficking, production, importation/exportation), among individuals under the age of 18, as well as consumption on the premises of schools and child-care facilities, airports, and on Canadian Coast Guard vessels and helicopters. The use of illegal drugs also remains prohibited on private property, including shopping malls, bars, and cafes [1].

In response to decriminalization, citing concerns around public safety, many BC communities implemented or expressed the intention to implement their own public consumption bylaws, further limiting where PWUD can use drugs in the community. Public consumption bylaws are a means by which communities can tailor regulations to their specific needs and values, and can be used to prohibit certain activities within a municipality with the aim of protecting public safety and controlling public behavior [3]. Acknowledging increasing concerns related to public consumption, Health Canada made additional amendments to the decriminalization policy on September 18th 2023. This update prohibited the possession of illegal drugs within 15 m of parks, spray or wading pools, or skate parks, province-wide [1]. Further, on November 9th, 2023, the provincial government of BC tabled additional legislation, *Bill 34*, ‘Restricting Public Consumption of Illegal Substances Act’, which outlines additional rules regarding drug use in public spaces across the province [4]. However, the implementation of *Bill 34* was placed on hold by the BC Supreme Court due to a constitutional challenge, citing concerns about its potential to cause harm to PWUD once in effect [5]. Most recently, on April 26, 2024, following an unsuccessful attempt to appeal the court’s pause on *Bill 34*, the BC government announced that they are in the process of requesting an additional amendment to the decriminalization policy by Health Canada to exclude consumption of drugs in all public places, thereby expanding the existing list of banned public spaces [6]. Their action plan includes new measures to provide police with more tools to address extraordinary circumstances where public safety is compromised, while simultaneously investing an additional $25 million to expand the provision of treatment and supports [6].

Considering that decriminalization seeks to alleviate the legal impacts associated with the policing of substance use, legislation and bylaws related to public consumption have the potential to result in unintended consequences for PWUD. This commentary will describe BC’s decriminalization policy, public consumption legislation, community bylaws, and reflect on the interplay of these policies. The commentary will discuss the potential effects of bylaws and public consumption legislation on communities, and its impact on the health and safety of PWUD, substance use, access to services, and efforts to reduce stigma, including its effects on marginalized populations. It will underscore the importance of comprehensively examining such legislation to understand its impact on decriminalization goals and marginalized populations.

## The context of decriminalization in BC

Historically, the legal framework for drug control and enforcement in Canada has been premised on prohibition, which forbids the possession, distribution, and production of any illegal substances without authorization [7]. Understanding the historical and colonial implications of prohibition is crucial for comprehending contemporary substance use policies, especially regarding public consumption bylaws.

The enactment of the prohibition of substances in Canada in the early 1900s, notably targeting substances like tobacco and alcohol, has had effects on societal attitudes and legal frameworks surrounding substance use [7, 8]. This prohibitionist framework has disproportionately impacted marginalized populations, leading to differential treatment in terms of enforcement and criminalization among these populations [7, 8]. Over time, Canadian drug policies have evolved to move away from prohibitionist approaches towards policies that recognize drug use as a public health issue rather than solely a criminal justice concern [9]. As such, decriminalization offers an opportunity to continue to push a public health-centred approach which can mitigate the harms associated with drug use.

Despite policy evolution, criminal drug laws continue to disproportionately impact marginalized populations and reinforce oppressive colonial power structures and systemic racism. For instance, drug law enforcement has led to mass incarceration, particularly among Black and Indigenous Peoples, who face higher rates of prosecution and incarceration for drug offenses compared to other communities [10]. When examining drug policy reforms in Canada, it is therefore essential to consider these historical and ongoing disparities.

Further, to understand how drug policies and related legislation are enacted in Canada, it is essential to understand the distinct roles of Canada’s various government levels. In Canada, there are three levels of government: federal, provincial, and municipal, each with its own distinct set of legislative powers. The federal government holds authority over matters of national concern, including the regulation of controlled substances through legislation like the CDSA, which generally prohibits activities involving controlled substances unless permitted by regulations or exemptions [9, 11]. However, provinces have the autonomy to enact additional laws and regulations, such as provincial bills aimed at preventing public consumption of substances [11]. Moreover, municipalities possess the authority to establish local-level bylaws that further regulate activities within their boundaries, including restrictions on public consumption of substances (e.g., tobacco, alcohol, as well as the drugs included under the CDSA exemption) [3]. This decentralized system allows for a layered approach to lawmaking, where municipalities can enact bylaws that complement, contradict, or extend provincial laws while adhering to the framework established by federal legislation [9, 11].

As a result, BC’s drug policy landscape is intricate. Illegal drugs are decriminalized provincially under the federal CDSA exemption. The BC provincial government oversees policies governing public consumption of these drugs (through *Bill 34*, for example). However, local municipalities retain the authority to enact their own bylaws, which can add further restrictions or regulations on drug use [11].

These historical and legal contexts set the backdrop for the current drug policy situation in BC, the implications of which are further described below.

## Provincial legislation restricting public consumption of illegal substances act

*Bill 34* proposes the prohibition of any public consumption of drugs within a 6-m radius from additional public spaces not initially included in the decriminalization policy amendment, including building entrances and public transit stops, and within 15 m of parks, beaches, and sports fields in all communities within BC [4]. The bill aims to allow police officers to direct anyone caught consuming an illegal drug in these specified areas to leave the area, and they will have the authority to arrest individuals for non-compliance and/or seize and destroy any illegal substances they may be using or carrying, even if under the 2.5 g threshold [4].

The Bill also provides municipalities with guidance on requirements for implementing bylaws in their respective communities, should they wish to impose any further public consumption-related bylaws [4]. The Bill states that municipalities wishing to do so will need to consult with the regional health board and the medical health officer responsible for public health measures within the area before considering a proposed bylaw [4].

As *Bill 34* currently faces an injunction, the government has indicated its intent to amend the decriminalization policy to re-criminalize drug use in all public spaces. Both of these initiatives, if passed, will effectively ban the public use of drugs province-wide.

## BC municipal bylaw legislation

Historically, BC municipalities have implemented various bylaws prohibiting the use of substances such as alcohol and smoking/vaping on public property, in public indoor areas, in public parks, some municipal properties, and on school grounds. After the federal legalization of non-medical cannabis in Canada in 2018, many of these municipal bylaws were updated to extend place-based restrictions to include the use of cannabis [12]. In the months prior to the tabling of *Bill 34*, a number of BC municipalities moved to adopt or amend existing local bylaws in order to regulate the use of illegal substances in public spaces such as municipal parks and facilities, streets, malls, or cafes, citing concerns over the safety risk to their communities. For instance, in January of 2023, several days before the decriminalization policy officially took effect, the City of Campbell River implemented a new bylaw prohibiting the open consumption of drugs on city property, including those listed under the decriminalization exemption [13]. This included a fine of up to $200 for violating the bylaw. This move was quickly appealed by a legal advocacy group, Pivot Legal Society, who launched a court challenge against the city on the grounds that it was outside of their jurisdiction to pass public health measures without consulting any public health officials [14]. In April 2023, the city made a second attempt to pass the bylaw, limiting its scope by outlining specific city-owned areas where the bylaw would apply, such as in specific municipal public spaces where children and families carry out leisure activities, and it was officially adopted on July 20, 2023 [13]. Instead of issuing a fine, the city will manage enforcement of the bylaw through education and redirection approaches for a trial period of 6 months, in which individuals will be connected to Campbell River’s only Overdose Prevention Site (OPS) [15].

Several municipalities followed suit. Between July and September 2023, the cities of Port Coquitlam, Pitt Meadows, Kamloops, Sicamous, and Nelson adopted amendments to their existing public consumption bylaws for alcohol and smoking to include substances listed under the decriminalization exemption [16–20]. Within the same time period, other municipalities, including Fort St. John, Maple Ridge, and Penticton, made attempts to propose similar bylaws, banning the use or display of illegal drugs or drug paraphernalia in their parks and public spaces [21].

## Potential implications of public consumption and bylaw legislation

Public consumption legislation carries the potential to bring about both benefits and costs for the community and PWUD. For instance, these laws carry the potential to promote community health and minimize the direct and indirect harms associated with public substance use, including neighbourhood disturbances, drug-related crime, violence associated with unregulated use, and stigma against PWUD [22]. However, they also hold the potential to undermine the decriminalization policy by re-entangling PWUD within the criminal justice system, reinforcing stigmatizing attitudes, and exposing them to substance use-related risks. This could potentially hinder decriminalization’s progress and lead to unintended consequences.

### Potential implications of public consumption and bylaw legislation for community health, public safety and stigma

The public consumption of psychoactive substances—legal or illegal—can pose risks to public health and community safety, while also reinforcing stigmatizing attitudes against drug use and PWUD. For instance, the disposal of drug-related paraphernalia and litter in public spaces may be associated with public consumption, posing risks of accidental injury and disease transmission to community members [22]. It can also lead to unsafe consumption or injection practices, endangering PWUD and increasing the risk of transmitting blood-borne viruses such as HIV and Hepatitis C [23, 24]. Furthermore, public drug use is often associated with drug-related crimes, as individuals seeking drugs may engage in survival crimes to support their use, such as theft, robbery, property crimes, and assaults [22]. Therefore, legislation regulating public consumption can help reduce unsafe use practices and improve public safety. Legislation governing substance use can also reduce exposure to second-hand smoke, protecting non-users from the harmful effects of passive inhalation. Drawing from global experiences with tobacco control policies, measures such as creating smoke-free public spaces have been highly effective in improving cardiovascular and respiratory health outcomes related to second-hand smoke exposure [24]. As such, policies regulating public consumption can mitigate the risk of second-hand harms from others’ drug use, and can contribute to increased feelings of safety and public amenity among community members [22].

Additionally, public consumption regulations may also significantly benefit individuals who may feel discomfort or face triggers in environments where they may be exposed to drug use, including those in recovery from drug use and individuals who have experienced harm or trauma associated with other’s substance use [25]. Limiting exposure to drug consumption in common public spaces has the potential to promote a safer and more inclusive environment for these individuals, which is crucial to their ongoing recovery and well-being [25].

However, touting public consumption legislation as a policy aimed at community safety may inadvertently reinforce the notion that legislation is necessary to safeguard communities from PWUD and to prevent public drug use. This narrative can have significant impacts on the overarching goal of decriminalization, which is to reduce the stigma associated with drug use. Laws clamping down on public drug consumption can potentially send a message to the public about the social acceptability of using drugs. Associating public drug consumption with inherent danger or risk to the public can stigmatize PWUD, reinforce negative stereotypes, and push them further into the margins of society. This may contradict the broader intent of decriminalization by reinforcing prohibitionist drug policies that emphasize the perception of drug use as a criminal rather than a public health issue.

Moreover, the specific stipulations of *Bill 34*, particularly its restrictions on illegal drug use within 15 m of parks, sports fields, playgrounds, and beaches, broadens the scope of areas where the consumption of illegal drugs is prohibited. This expansion is in contrast to the current provincial regulations for other regulated substances such as alcohol, tobacco, and cannabis, which only prohibit their use directly within these spaces, not in the surrounding vicinity [12]. Further, it contradicts recent amendments made to alcohol bylaws in some BC communities such as Vancouver, which permit drinking in a growing number of public areas, including parks and beaches, in addition to regulated areas such as bars and restaurants, underscoring the disparate policy approach that has been taken in regards to these regulated substances [26]. These differences have the potential to further stigmatize drug users, suggesting that there is no place for their consumption within and around such public places. These contrasting approaches highlight disparities in the social acceptance of different types of drugs, disproportionately stigmatizing the users of some drugs over others.

This stigma can have damaging effects, leading to social isolation, alienation, increased risk of using alone, and the creation of an uncomfortable and hostile environment that discourages PWUD from accessing critical support services, such as housing, employment, harm reduction, treatment, and social services [27–29]. When individuals are deterred from accessing critical supports due to stigmatization, their ability to engage with life-saving programs and services diminishes. Furthermore, experiences with internalized stigma have been extensively documented as barriers to help-seeking among PWUD [27, 29]. Taken together, these stigmatizing perceptions have the ability to significantly impact the decision-making of PWUD as to whether they seek service support.

### Potential implications of public consumption and bylaw legislation for risks related to drug use initiation and harms

Public consumption legislation aimed at discouraging drug use in specific public areas may have broader implications beyond public and community safety, including in relation to drug use initiation and drug use-related harm.

Regulations that discourage drug use in certain public areas may hold the potential to reduce the likelihood of substance use initiation within communities. For instance, existing evidence suggests that public policies regulating tobacco consumption play a significant role in deterring young individuals from starting to use tobacco and in facilitating smoking cessation among both adults and adolescents [23, 24]. The same may be true about alcohol [30]. This is because such policies can reduce the visibility of and exposure to substance use, subsequently de-normalizing and diminishing the social acceptability of substance use among individuals, particularly youth [23].

However, while recognizing the potential to discourage substance use initiation, public consumption bylaws may lead to riskier drug use practices. Such legislation can displace individuals to spaces where they are more likely to use drugs alone or in secluded areas, which can lead to increased health risks, such as unsafe drug use practices or overdoses [27]. In these hidden settings, there is often no one present to provide immediate assistance or administer life-saving naloxone in case of an overdose. Emergency response times may be delayed, heightening the chances of a fatal overdose [27]. The data on unregulated drug deaths from the BC Coroners Service revealed that in 2023, 80% of these tragic fatalities occurred indoors, 48% of which occurred in private residences, and another 32% in various other residential environments, such as supportive housing, shelters, hotels, and single room occupancies, as well as in businesses and public buildings [31]. These data underscore a pressing need to create environments where PWUD feel safe accessing harm reduction services, rather than resorting to using drugs in private spaces, especially in light of public consumption restrictions.

Public consumption legislation may further encourage expedited drug use behaviors by making PWUD feel that they need to use their drugs as quickly and discreetly as possible when using in public to avoid criminalization, placing them at even greater risk [32]. Hasty drug use in an effort to avert criminalization or the public’s gaze can lead to practices such as rushed injections or improper hygiene procedures, which can also increase the risk of developing abscesses. In these circumstances, PWUD may overlook proper sterilization of consumption and injection equipment, and can result in environmental hazards, such as discarded needles and paraphernalia, endangering the broader community [32]. Additionally, under *Bill 34*, punitive policing practices such as drug seizures may be enforced in public spaces, which could lead to increased interactions with the unregulated drug market among PWUD [33]. PWUD who face the risk of seizures of their substances may be forced to seek alternative and perhaps unfamiliar sources to replace what has been confiscated, which has the potential to increase the risk of fatal or non-fatal overdose from a potentially contaminated drug supply [33]. Therefore while public consumption bylaws and legislation may reduce the likelihood of drug initiation, they may also result in the unintended consequences of increasing risk for drug-related harms.

### Potential implications of public consumption and bylaw legislation for accessing housing and harm reduction services

Under bans on the public consumption of drugs, PWUD will increasingly have to rely on access to indoor spaces such as private homes or harm reduction services such as supervised consumption services (SCS) and overdose prevention services (OPS) to consume their drugs. SCS and OPS offer safe and controlled spaces for drug consumption under the supervision of professionals and peers trained in overdose prevention, and provide harm reduction supplies, medical assistance in case of overdoses, and other key services such as drug checking, safer supply programs, and referrals to other services [34–36]. However, there is a paucity of both housing and SCS/OPS available in communities throughout the province, which makes it difficult for PWUD to find safe and accessible spaces to consume drugs [37]. Therefore, it is crucial that public consumption regulations are accompanied by enhanced access to housing and SCS/OPS services to ensures that PWUD have access to safe and supportive environments to consume their drugs.

The ongoing housing crisis in BC highlights significant challenges in addressing the social and structural factors that contribute to drug use. A lack of access to secure housing is compounded by housing unaffordability and a limited availability of low-barrier housing programs and supports in the province that do not require PWUD to be abstinent as a precondition for eligibility [38]. Without stable housing, individuals may face increased exposure to drug use in unsafe environments, an elevated risk of overdose due to using alone or in secluded areas, and risks related to being subject to re-criminalization [38]. Addressing housing insecurity is therefore crucial in mitigating the adverse consequences of drug use and promoting the health and well-being of PWUD [39–41].

In addition to a lack of housing, there is also a lack of harm reduction services in BC, with only 44 OPS and 4 federally-sanctioned SCS currently in operation [37]. This limited number underscores significant gaps in the ability of PWUD to access these essential services, resulting in the potential for re-criminalization when using outdoors. Increasing the number of and access to SCS and OPS would not only address concerns surrounding public consumption but could also help mitigate issues related to the disposal of drug-related paraphernalia in public areas and other public nuisance concerns. It could also contribute to reducing the incidence and presence of drug-related crimes [40, 41].

Additional community bylaws and zoning restrictions exist within many municipalities, further exacerbating challenges with SCS and OPS service implementation. For instance, some regulations restrict land use and development within a given area, whereas others restrict smoking and vaping indoors and within specified distances of outdoor spaces such as parks, playgrounds, and entryways [42]. These bylaws may present specific challenges, including an inability for SCS/OPS to offer inhalation services for PWUD [42]. Smoking is currently the most common form of illegal drug consumption in the province, and has increased from 29% to 56% between 2016 and 2021, while injection has declined from 39% to 20% within the same time period [31]. However, despite the preference for inhalation as a mode of consumption for many PWUD, as of September 2023, only 42% (or 20 of the 48 sites across the province) offer a safe place for people to smoke [37]. The inability of SCS and OPS to offer inhalation services may undermine the decriminalization policy, which aims to reduce barriers to life-saving services and supports to prevent health harms [2]. Investments in expanding housing and harm reduction services for PWUD and providing safe spaces for drug use away from the public can combat ‘Not In My Backyard’ mentalities (NIMBYism), in which people resist initiatives like SCS or OPS in their local communities [43]. Increasing access to these services for PWUD can reduce community stigma around drug use and ultimately mitigate NIMBY attitudes that often hinder the implementation of and support for harm reduction services [34]. When communities see drug use as a health issue and observe the benefits of harm reduction services, such as fewer overdose deaths and less public drug use, they may become more accepting of these services, potentially combatting stigma [34].

### Potential implications of public consumption and bylaw legislation for vulnerable populations

It is crucial to recognize that restrictions on public drug consumption may have particularly consequential implications on vulnerable populations, including individuals experiencing homelessness, who often lack alternatives and may be forced to use drugs in public settings. Historically, marginalized and racialized populations, who experience higher rates of visible poverty and housing precariousness, are much more likely to be criminalized for the consumption of drugs in public spaces [44]. These populations have been largely excluded from the development of population-level drug use policies such as public consumption regulations, and are more at risk of experiencing the harms and implications associated with the application of these laws [44, 45]. For instance, as briefly described above, the City of Vancouver passed a recent bylaw in 2023 that allows public drinking in several city parks, however, parks located near and within Vancouver’s Downtown Eastside, a neighbourhood with high incidences of drug use overdoses, criminalization, and poverty, and home to 31% of the city’s Indigenous population, have been largely excluded from the program [45, 46]. As such, residents in this vulnerable neighbourhood continue to be targeted by over-policing and the inequitable application of these bylaws.

Additionally, recent data reveals a striking 32% increase in homelessness in Vancouver in 2023 compared to 2020, with 71% of those individuals reporting addiction [47]. People experiencing homelessness face a multitude of challenges, especially amid the housing crisis, with limited shelters and housing services. Without access to designated drug-use spaces they face heightened vulnerability to harassment, including from law enforcement, and risk drug confiscation due to having to carry their entire supply [32, 41]. This exacerbates housing insecurity, potentially perpetuating cycles of poverty and substance use. As a key example, in Prince George, the controversial Safe Streets bylaw enacted in August 2021 imposes fines ranging from $100–$50 000, imprisonment, and other penalties for activities like open drug use and panhandling [48]. Results from a preliminary analysis conducted by the British Columbia Assembly of First Nations in 2022 found that 40% of respondents felt less safe, and 62% found it harder to consume drugs safely due to bylaw officers confiscating harm reduction supplies [48]. The punitive nature of the bylaw, combined with inadequate public education, increased inequities among PWUD and homeless populations, and strained city resources has increased petty survival crimes and contradicts public safety goals [48].

Public consumption legislation represents strategies for drug control that perpetuate the historical colonial nature of prohibitionist frameworks. This approach contradicts the goals of decriminalization, which seeks to dismantle such frameworks and promote public health initiatives. Depending on their design and implementation, these policies can perpetuate inequalities among oppressed and marginalized groups [10, 49]. The current design of public consumption laws, does not effectively address the root causes and social determinants of drug use and public consumption. Consequently, they may lead to increased police interventions and PWUD interactions with the criminal justice system, as well as more arrests of marginalized individuals for public consumption offenses [10, 49].

## Support and opposition of public consumption legislation and bylaws

In line with the potential benefits and costs of public consumption legislation described above, several organizations have taken various positions either in support or opposition to these bylaws. For instance, public consumption legislation has garnered political and community support due to its role in upholding public order and safety. Some political leaders have highlighted the law’s perceived potential to enhance community well-being, while others have suggested that the legislation equips law enforcement with vital tools for fostering public safety, all while emphasizing a non-criminal approach to guiding drug users toward care pathways [50].

However, several organizations (e.g., the Harm Reduction Nurses Association Vancouver Area Network of Drug Users, Canadian Mental Health Association BC Division, BC Association of Social Workers, the BC Green Party, and Union of British Columbia Indian Chiefs) have publicly opposed the implementation of *Bill 34*, expressing concerns about its potential impact on the health, safety, and fundamental rights of PWUD [51–57]. In particular, arguments have emphasized the specific implications of the bill on unhoused individuals, calling for strategies to address issues surrounding the lack of affordable housing and other supports [53–55]. Collectively, these organizations maintain that there is a lack of evidence demonstrating any association between increased public drug use and decriminalization, and argue that the bill and community bylaws re-criminalize drug using communities which may drive drug use underground, increasing risks and fatalities, undermining the objectives of decriminalization [51–57].

## Recommendations for next steps

### Engage multidisciplinary stakeholders including PWUD in decision-making

Going forward, in order to fully achieve the goals of the decriminalization policy, the province should consider the potential impacts of public consumption legislation, such as *Bill 34* and community bylaws. The design and implementation of policies that directly impact PWUD necessitate the active engagement of those directly affected. However, a significant issue that has emerged in the discussions surrounding public consumption legislation is the perceived exclusion of organizations and advocacy groups representing PWUD [52]. These voices, which are essential in shaping policies that directly impact PWUD, appear to have been marginalized in the decision-making process. As described above, advocacy groups and representatives for PWUD and other marginalized communities have publicly opposed such legislation, urging for a more comprehensive, non-punitive approach to address the underlying challenges driving drug use and public safety concerns in BC [51–57]. Involving PWUD and other historically marginalized or racialized communities, advocacy groups, and frontline workers can ensure that policies do not perpetuate harmful inequities caused by previous prohibitionist drug strategies, are rooted in real-world experiences, and are responsive to evolving needs and concerns [58].

The legislation’s potential implementation must be thoughtfully approached to ensure it aligns with the principles and objectives of the decriminalization policy, and appropriately considers the unique realities and perspectives of everyone they aim to benefit [58]. Otherwise, it runs the risk of further contributing to existing inequities.

### Expand harm reduction and housing services

It is crucial to address its underlying structural factors related to homelessness, vulnerability, and the lack of safe spaces for PWUD. Focusing on these systemic challenges first and developing viable solutions should be a priority over enacting public consumption legislation. By ensuring that individuals have access to stable housing, comprehensive support services, and harm reduction resources, a foundation can be created upon which public consumption legislation can be considered. Introducing such legislation without concurrently addressing these systemic issues may not only be ineffective but is also unjust to PWUD as it fails to provide them with appropriate alternatives and support systems.

Expanding housing and social supports, along with enhancing accessibility to essential harm reduction services such as OPS and SCS, is urgently needed. Research has consistently indicated that improving access to private indoor spaces, such as low-threshold supportive housing and consumption sites, can reduce public disorder and drug-related litter, decrease interactions with law enforcement, and minimize the potential harms from open drug scenes for both the public and PWUD [40, 41]. Moreover, the integration of harm reduction and substance use treatment services within housing-first models, which prioritize permanent, low-barrier housing without prerequisites for abstinence or treatment has been associated with improvements in health and social well-being outcomes, such as employment and higher retention in care [59–61].

To expand harm reduction services effectively, establishing supervised inhalation sites is crucial. Since inhalation is a preferred consumption method for many PWUD, especially with the potential of public consumption laws in place, these sites are vital. Currently there are no indoor stand-alone supervised inhalation sites in the province. Instead, most operate via temporary setups like covered outdoor tents, garages, and trailers, as permanent SCS and OPS locations often lack the space and ventilation infrastructure to be able to provide inhalation services [62].

Adding to the complexity of the issue, some municipal representatives have cited perceived limitations in their authority to open up such services, frequently pointing to provincial regulations that prohibit indoor smoking, as well as other economic and occupational health and safety concerns [62]. These regulatory challenges align with smoking and zoning bylaws that similarly restrict indoor smoking and the use of land for OPS purposes, creating a web of interconnected issues and contributing to the confusion surrounding the implementation of vital harm reduction services. However, temporary measures can be adopted to address this issue whilst promoting broader acceptance of permanent solutions amidst the evolving discourse on drug use under decriminalization. For instance, the introduction of mobile SCS in several BC cities offers a feasible interim solution that may be more readily accepted by the public [34]. Furthermore, applying for municipal council resolutions to temporarily lift indoor smoking bans at SCS/OPS for a trial period can be explored [63]. This approach allows for monitored indoor inhalation, while assessing its effectiveness in practice.

The investment in social and harm reduction services to provide spaces for PWUD to access and help address the systemic issues they face requires financial commitment. Recently, the BC government has substantially focused on expanding treatment and recovery services, with $586 million (59%) of the $1 billion allocated for mental health and addictions support in the 2023 provincial budget going towards increasing treatment and recovery beds for PWUD [64]. These investments are welcome, and should be accompanied by investments in other pillars of the overdose crisis response, including poverty reduction and the expansion of evidence-based harm reduction services. Harm reduction and housing-first strategies are suggested to be cost-effective approaches for healthcare systems to help prevent and reduce the harms associated with drug use, especially when combined with evidenced-based treatments [39, 65–67].

By pairing public consumption restrictions with investments in secure housing and other indoor consumption spaces like OPS and SCS, communities can aim to strike a balance between what they deem as public safety concerns and the welfare of PWUD.

### Conduct thorough evaluations

The importance of conducting ongoing comprehensive evaluations to assess the impacts of public legislation and bylaws on PWUD and the decriminalization policy cannot be overstated, as it holds significant implications in several critical areas. Firstly, it is essential to gauge whether these policies reduce or, conversely, increase stigmatization against PWUD. The shift towards decriminalization is grounded in recognizing drug use as a public health issue rather than a criminal one. Therefore, if the evaluation reveals an increase in stigmatization, it signals a misalignment between the policy’s intentions and its real-world consequences. However, if the evaluation reveals a decrease in stigmatization, it may suggest that the policies are effectively changing public perceptions and attitudes toward PWUD.

Secondly, assessing whether these policies result in an increase in risky drug use practices is critical. Decriminalization aims to reduce the harm associated with drug use. However, if the evaluation shows an uptick in dangerous drug consumption patterns, it may indicate a need for enhanced harm reduction measures and services. This information will be vital to refine policies and ensure they effectively protect the health and well-being of PWUD.

Additionally, evaluating the legislation’s impact on the utilization of harm reduction services, is of paramount importance. An increase in the availability and use of these services could signal the legislation’s effectiveness in providing safe spaces for PWUD to consume drugs. However, if the services remain unchanged in terms of increasing capacity and accessibility, which can be coupled with lack of increase in utilization and access, it can signify the need for additional supports. This aspect of evaluation is especially crucial for people experiencing homelessness, as it directly affects their access to vital harm reduction resources and services.

The implementation of public consumption legislation within a short timeframe after the decriminalization policy raises concerns about the sequence of policy actions and their overall effectiveness. Although it is unclear what will influence decision-making with regards to the currently proposed public consumption legislation, it is also important to note that the potential of introducing this legislation in such close proximity to the implementation of the decriminalization policy can pose challenges in appropriately assessing and evaluating its impact. A critical aspect of a comprehensive evaluation is the need for sufficient time to gather meaningful data and analyze trends while minimizing confounding factors. With the rapid introduction of public consumption legislation, there may not be adequate temporal distance to discern the nuanced effects of the decriminalization policy.

Overall, conducting a thorough evaluation is crucial to safeguarding the integrity of public consumption legislation and bylaws as it relates to decriminalization. It enables policymakers to make data-driven decisions that align with the core principles of harm reduction and public health and consistently reflect on-the-ground realities, ensuring that PWUD receive the support and dignity they deserve.

## Conclusion

The relationship between the decriminalization of illegal drugs in BC and policies related to the consumption of illegal drugs in public is complex, and many questions remain to be answered, including how the potential implementation of the tabled public consumption legislation will impact the decriminalization initiative. Banning public consumption may hold some benefits. However, it also has the potential to undermine the policy, reinforce inequities and harms associated with drug prohibition and the criminal justice system, and entrench stigma regarding drug use, pushing PWUD to engage in risky behaviours, increasing risks for harms such as infections and overdoses, and creating barriers for engaging with health and treatment services. Going forward, it will be important to conduct further consultations with PWUD and advocacy groups and monitor and evaluate the potential effects of public consumption and bylaw legislation, to ensure that these do not negate the spirit of decriminalization and result in unintended consequences.

## Data Availability

Not applicable.

## Abbreviations

BC: British Columbia
CDSA: Controlled Drugs and Substances Act
NIMBY: Not In My Backyard
OPS: Overdose Prevention Site(s)
PWUD: People who use drugs
SCS: Supervised consumption service(s)
